# Obstetrics; the Science and the Art

**Published:** 1849-07

**Authors:** 


For sale by G. H. Derby & Co.

---

*Obstetrics; the Science and the Art*; by Charles D. Meigs, M. D., Prof. of Midwifery, etc., in the Jefferson Medical College at Philadelphia, etc., etc., etc. With one hundred and twenty-one illustrations. Philadelphia: Lea & Blanchard, 1849, 8 vo. pp. 683.

The deservedly high reputation of Prof. Meigs, as a practitioner and teacher of Obstetrics, his varied scientific and scholastic acquirements, and his vast experience, will ensure for this new volume a prompt and cheerful appreciation by his American brethren. With his former treatise on the same subject, entitled the "Philadelphia Practice of Midwifery," our readers are, of course, familiar. The second and last edition of that popular work has been for some time exhausted. The present work, which is designed to take its place, is much more comprehensive in its scope, and embraces the fruits of a more extended period of observation and research.

The eccentricities of composition in the writings of Prof. M., have given some occasion for criticism. His excessive predilection for classical, foreign, far-fetched, and newly coined terms, sometimes renders his style pedantic, and, for not a few of his readers, obscure. His letters on the diseases of females, particularly, were open to critical objections on this score. The present work, we are glad to see, is written in far better taste, in this respect. Indeed, not only may it be read without the necessity of having several dictionaries within reach, but the style, so far as we are capable of judging from slight examination, is simple, chaste, and consequently clear and agreeable.

As regards the scientific and practical value of the work, we have only to say, that whatever Prof. Meigs may publish concerning the principles and practice of Obstetrics, will amply repay careful perusal by the medical student and practitioner. To say this is supererogatory. The bare announcement of the work, would be sufficient to suggest and commend to to the judgment of our readers the truth of the remark. We should add, that, in mechanical execution, the work is in the excellent style which characterizes the medical publications emanating from the house of Lea & Blanchard. For sale by G. H. Derby & Co.

